# Comprehensive Analysis of the Influence of Fulvic Acid from Paper Mill Effluent on Soil Properties, Soil Microbiome, and Growth of *Malus hupehensis* Rehd. Seedlings under Replant Conditions

**DOI:** 10.1021/acsomega.1c03201

**Published:** 2021-09-10

**Authors:** Xiaoqi Wang, Yuanyuan Yao, Guiwei Wang, Jinzhao Ma, Chengmiao Yin, Xuesen Chen, Zhiquan Mao

**Affiliations:** † College of Horticulture Science and Engineering, State Key Laboratory of Crop Biology, Shandong Agricultural University, Tai’an 271018, China; ‡ National Engineering Laboratory for Efficient Utilization of Soil and Fertilizer Resources, College of Recourses and Environment, Shandong Agricultural University, Tai’an 271018, China; § College of Resources and Environmental Sciences, China Agricultural University, Beijing 100193, China; ∥ Shandong Provincial Key Laboratory of Eco-Environmental Science for Yellow River Delta, Binzhou University, Binzhou 256603, China

## Abstract

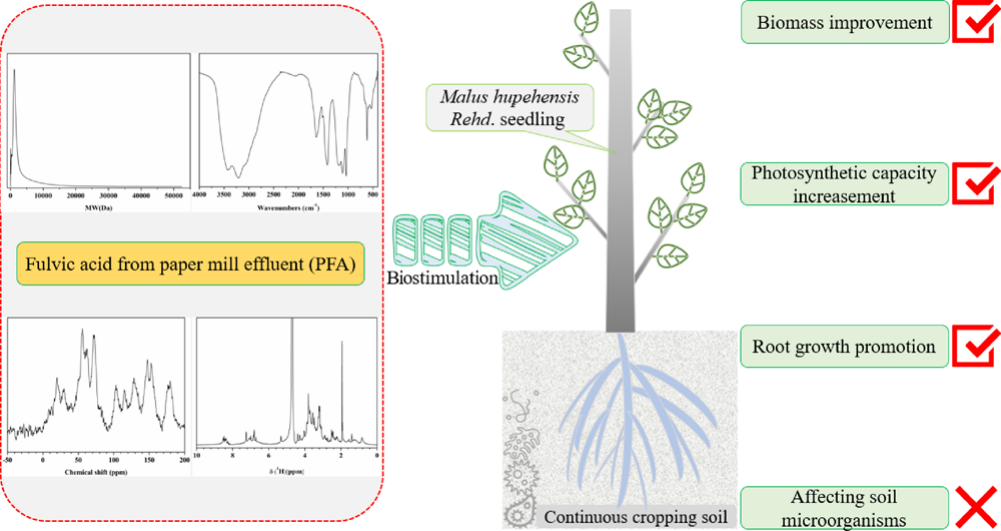

In this study, the potential regulatory effects of fulvic acid extracted from paper mill effluent (PFA) in apple replant disease (ARD) were investigated through a comprehensive experimental evaluation of the effects of PFA on soil properties, growth inhibition of apple replant pathogens, and growth of replanted *Malus hupehensis* Rehd. seedlings. PFA with a relatively lower molecular weight was mainly composed of carbohydrates, lignin derivatives, and polysaccharides and was rich in functional groups such as carboxyl and phenolic hydroxyl groups. Treatment with PFA dosages ranging from 2 to 3 g/pot significantly increased available phosphorus (P) in soil by 47.5 to 57.5% when compared with the control without PFA, indicating that PFA had a positive effect in activating P. In addition, PFA stimulated the growth of replanted seedlings by promoting root elongation, enhancing leaf photosynthesis, and increasing the activity of root antioxidant enzymes including superoxide dismutase, peroxidase, and catalase. However, no convincing evidence was found that application of different dosages of PFA had remarkable effects on soil pH, inorganic nitrogen, available potassium, organic matter, and the numbers of bacteria and fungi. Notably, PFA had no effect on the copy number of the main pathogenic fungi causing ARD, including *Fusarium oxysporum*, *Fusarium solani*, *Fusarium proliferatum*, and *Fusarium moniliforme*. Overall, PFA can alleviate ARD to a certain extent mainly through its effects on improving the resilience of replanted young seedlings rather than by affecting soil microorganisms or providing nutrients.

## Highlights

- Apple replant disease (ARD) can be partly mitigated by the application of fulvic acid extracted from paper mill effluent (PFA).
- PFA exhibited a positive effect on root development and leaf photosynthesis, as well as the protective enzyme activity of the replanted seedlings.
- PFA alleviated ARD mainly through its biostimulation rather than by increasing the nutrient supply or affecting soil microorganisms.

## 1 Introduction

China ranks as the largest apple producer and consumer in the world with a planting area and an annual output accounting for 2.22 million hectares and 41.39 million tons, respectively.^1,2^ Most apple orchards in China were established in the 1980s and 1990s, and due to the aging of fruit trees, the acceleration of the optimization of tree species, and popularization of dwarfing rootstocks, the renewal of orchards is becoming increasingly frequent. One of the major challenges of orchard renewal is the inhibition of plant growth caused by apple replant disease (ARD),^3^ especially in the face of scarce stubble land. ARD can restrain root growth,^4^ slow down tree development,^5^ delay fruiting, and reduce productivity,^6^ thereby lowering the income of growers from $70,000 to $150,000 per acre during the initial 4 years and resulting in an economic loss of up to 50% in the entire life cycle of the orchard.^7,8^ Currently, the widespread ARD has become a common problem that seriously restricts the sustainable development of the apple industry in China.

ARD is a soil-borne disease caused by a complex of the imbalance of nutrient elements,^9^ soil property deterioration,^10^ allelopathy accumulation,^11^ and shifts in bacterial and fungal communities.^12^ Biotic factors, including explosive reproduction of harmful fungi and the deterioration of the microbial community structure after long-term planting of fruit trees, are recognized as the dominant factor resulting in ARD.^13,14^ In recent years, broad-spectrum fumigants, such as methyl bromide, which indiscriminately kill most soil microorganisms, have favorable effects in controlling ARD. However, due to the serious adverse effects of chemical fumigants on environmental pollution, ozone depletion, and potential hazards to human health,^15^ their application is no longer permitted in most countries. Thus, alternative green and low-cost treatments are urgently needed for ARD control.^16^

Plant biostimulants are defined as substances and products, whose function is to regulate physiological and biochemical responses in plants, enhance plant tolerance to abiotic/biotic stresses, and boost the growth and development of plants at a relatively low application rate.^17,18^ Biostimulants are classified into eight categories including humic substances, seaweed extracts, chitosan derivatives, complex organic materials, *etc*.,^19^ and have been widely used in agricultural production. For instance, some biostimulants, like chitosan derivatives, exhibited a positive effect in controlling ARD by improving seedling resistance, boosting root growth, and regulating the soil fungal community.^20^ At issue is whether other biostimulants have similar physiological effects to ameliorate the growth of replanted seedlings under continuous cropping conditions.

Fulvic acids (FAs) are a family of natural organic acids with broad-spectrum biostimulatory effects, whose beneficial roles in promoting plant growth and improving the soil properties have been well recognized.^21^ Currently, sources of FAs mainly originate from peats or coal, whose nonrenewable characteristic gives rise to a focal interest in utilizing sustainable organic wastes for commercial FA products. Recent studies noticed that the organic matter in paper mill effluent of the straw-based ammonium sulfite pulping process exhibited similar characteristic functional groups as well as biostimulatory activity to that in FAs, thus naming it as a “fulvic acid-like substance from paper mill effluent” (PFA).^22,23^ In general, FAs/PFA can not only increase the nutrient utilization efficiency of plants through its biostimulatory effects^24,25^ but also enhance the photosynthesis of plants by regulating the opening/closing of leaf stomata,^26^ thereby regulating the metabolism of plants to enhance plant resistance under adverse conditions.^27^ For the soil, active groups in PFA such as carboxyl and phenolic hydroxyl groups are important carbon energy sources, which are conducive to improving the physical properties of the soil.^28^ It has also been shown that PFA can accelerate the formation of macro/microaggregates by increasing the cohesive force between clay particles and fine particles when PFA is associated with the large surface area of soil.^29^ However, research studies on PFA to date have mainly focused on alleviating abiotic stresses such as salt,^22,23^ drought,^30^ or nutrients,^31^ but its effects on ARD that are dominated by biological factors remain unclear.

Accordingly, an experimental pot study was conducted with the aim to explore the mechanism of which PFA affects the replanted apple seedlings under continuous cropping conditions by evaluating the effects on soil properties, plant growth, and soil microorganisms. This research will help provide practical implications, open research avenues in the efficient application of PFA for controlling ARD, and provide an application prospect for paper mill effluent.

## 2 Results and Discussion

### 2.1 Characterization of PFA

**Figure 1:**
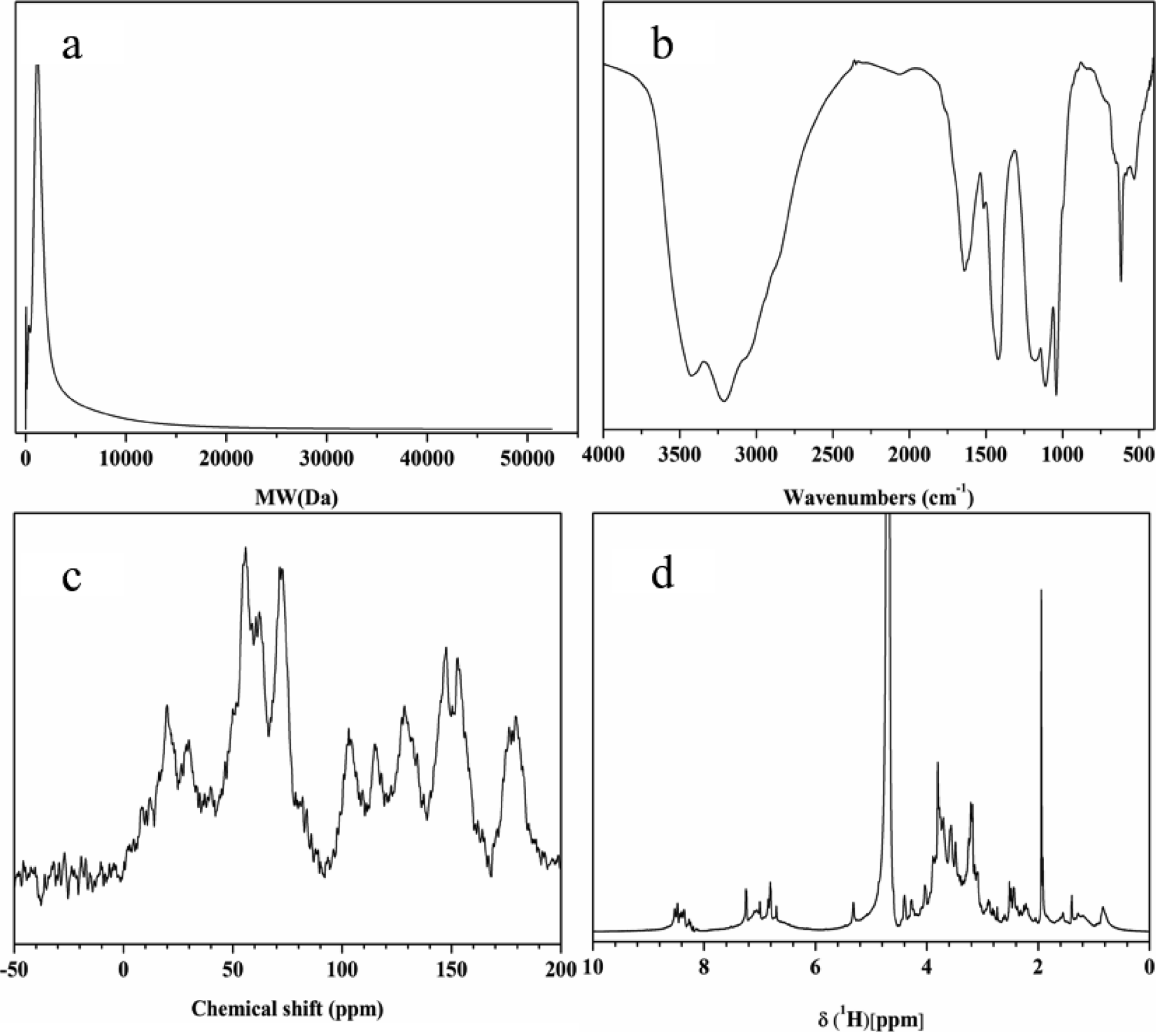
GPC (a), FTIR (b), solid-state ^13^C NMR (c), and liquid-state ^1^H NMR (d) of PFA.

The chemical structure of PFA is complex. Therefore, the molecular weight distribution and structure composition of PFA were analyzed by a series of characterization methods. Gel permeation chromatography (GPC) analysis indicated that the relative ratio of PFA with the molecular weight of less than 1000 Da is 19.4%, the fraction of 1000–5000 Da is 59.5%, and the remaining fraction is 21.1% (Figure 1a). Accordingly, PFA has a lower molecular weight than other biostimulants (usually up to 10^6^ to 10^7^ Da),^19^ and then it can easily penetrate the pores of cell membranes and be absorbed by plants.^32^ The Fourier transform infrared (FTIR) spectrum of PFA (Figure 1b) revealed a broad peak at around 3400 cm^–1^ that is usually produced by the stretching vibration of the O–H bond. In addition, the absorption peak present near 1718 cm^–1^ corresponds to the C–O vibration in the carboxyl group, which is also the typical absorption band of fulvic acid extracted from lignite.^33^ The band at 1515 cm^–1^ was the characteristic absorption peak of lignin, the absorption band at 1426 cm^–1^ was ascribed to the vibration of O–H in the carboxyl group, and the spectral band at 1042 cm^–1^ indicated that PFA contained a polysaccharide structure. In addition, the solid-state ^13^C nuclear magnetic resonance (^13^C NMR) spectrum (Figure 1c) and liquid-state ^1^H nuclear magnetic resonance (^1^H NMR) spectrum (Figure 1d) provided detailed structural information of PFA. The distribution of the ^13^C NMR spectrum confirmed that PFA contained more carbohydrates, a lignin-derived aromatic structure, and carboxyl groups. The proton interval distribution of the ^1^H NMR spectrum indicated that there were many alkyl protons, aromatic protons, and protons on the C atom, which directly combined with N and O. In summary, PFA is a low-molecular-weight biostimulant, has high contents of carbohydrates, lignin derivatives, and polysaccharides, and exhibits similar characteristic functional groups to those in FAs such as carboxyl groups and phenolic hydroxyl groups.^22,34^ Thus, it can be recognized as an alternative to the nonrenewable FAs.^22^

### 2.2 Effects of Different Amounts of PFA on Soil Properties

**Figure 2:**
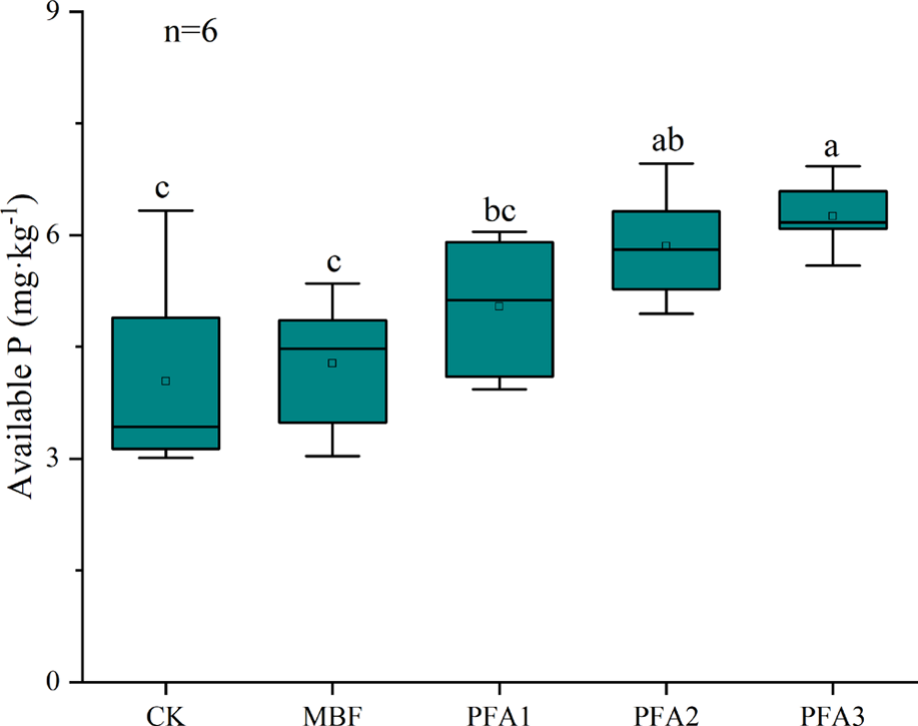
Soil-available P under different treatments.

This study revealed that methyl bromide fumigation or different dosages of PFA had no significant effect on soil pH, nitrate nitrogen (NO_3_^–^-N), ammonium nitrogen (NH_4_^+^-N), available potassium (K), and organic matter contents (Table 1). On the other hand, the application rate of PFA markedly affected the soil-available phosphorus (P) content (Figure 2). More concretely, soil-available P increased with increasing concentration of applied PFA, although no significant difference was observed between PFA1 and CK. The contents of available P in PFA2 were 47.5 and 37.2% higher than those in CK and MBF, respectively, and the content of available P in PFA3 was significantly increased by 26% compared with PFA1.

**Table 1:** Soil Physical and Chemical Properties under Different Treatments^a^

treatment	pH	NO_3_^–^-N (mg/kg)	NH_4_^+^-N (mg/kg)	available K (mg/kg)	organic matter (g/kg)
CK	7.10a	8.9a	4.1a	84.2a	5.9a
MBF	7.12a	9.0a	5.3a	82.3a	6.3a
PFA1	6.99a	8.6a	4.8a	75.2a	6.5a
PFA2	7.07a	8.1a	4.8a	74.5a	6.4a
PFA3	7.01a	7.9a	5.2a	75.4a	6.2a

a Note that means followed by the same lowercase letter within each column in the same year were not significantly different (*p* > 0.05) based on the analysis by one-way ANOVAs followed by Duncan multiple-range tests (*n* = 6).

The deterioration of soil properties caused by long-term planting is one of the major causes of ARD.^6^ After apple trees have been planted for a longer time, the soil physical structure can be seriously damaged, and this is accompanied with an increase in soil bulk density and a decrease in porosity.^6^ PFA was rich in carboxyl, phenolic hydroxyl, and other functional groups (Figure 1), and these functional groups were conducive in fixing elements in the soil by chelation, complexation, ion exchange, and potential adsorption, thereby promoting the formation of soil aggregates, ultimately improving the soil physical structure.^29^ It has been reported that PFA can enhance nitrogen (N) fixation by improving the soil buffer capacity, thus achieving high N use efficiency.^35^ However, no significant effect on soil inorganic N content by PFA was observed in the present study. With the increase in the PFA application rate, the content of soil-available P showed an upward trend, which may be attributed to the fact that the increased content of acid functional groups in PFA accelerates the release of P. In addition, the anions in PFA can compete with PO_4_^3–^ for specific adsorption sites on the solid surface to reduce the adsorption of soil P.^28^ In addition, PFA can form soil humic matter clay mineral complexes, thereby reducing P fixation. Since P is an important influencing and limiting factor in ARD control,^36^ it is speculated that PFA may activate the soil P in the long-term process, thus alleviating the ARD to a certain extent.

### 2.3 Effects of Different Amounts of PFA on the Growth of Replanted *Malus hupehensis* Rehd. Seedlings

Methyl bromide fumigation or PFA application significantly boosted the growth of replanted *Malus hupehensis* Rehd. seedlings. The plant height, ground diameter, aboveground biomass, and root weight of replanted seedlings in MBF, PFA1, PFA2, and PFA3 were significantly higher than those in CK (Table 2). However, only the ground diameter of PFA2 was significantly higher than those of PFA1 and PFA3. Well-developed roots can help improve the ability of plants to absorb nutrients and water.^20^ The root development status under different treatments also had a similar trend with the aboveground biomass. Methyl bromide fumigation or application of PFA at 1 and 2 g/pot significantly increased the total root length, root surface area, root volume, and average root diameter of the replanted seedlings (Table 3), thus partly ensuring the normal operation of physiological, biochemical, and metabolic reactions in plants. Wang *et al.*’s research also confirmed that PFA promoted root development by increasing the cell size and cell division.^24^ However, with the increase in PFA application rate, the elongation of roots showed an inhibitory trend; even the root surface area and average root diameter had no significant difference with the CK treatment when PFA was applied at 3 g/pot.

**Table 2:** Plant Growth Status of Different Treatments^a^

treatment	plant height (cm)	diameter (mm)	aboveground biomass (g)	root weight (g)
CK	20.4c	4.3c	6.7c	5.7c
MBF	42.1a	5.7a	17.7a	15.6a
PFA1	32.1b	4.4c	12.3b	10.8b
PFA2	32.6b	5.0b	12.9b	11.2b
PFA3	30.5b	4.4c	11.6b	10.3b

a Note that means followed by the same lowercase letter within each column in the same year were not significantly different (*p* > 0.05) based on the analysis by one-way ANOVAs followed by Duncan multiple-range tests (*n* = 6).

**Table 3:** Root Morphology under Different Treatments^a^

treatment	total length (mm)	surface area (mm^2^)	root volume (mm^3^)	average root diameter (mm)
CK	1884.7c	695.6c	10.7c	1.4c
MBF	3661.4a	1189.9a	15.7a	2.4a
PFA1	2394.2b	919.5b	12.5b	2.0b
PFA2	2434.8b	895.1b	12.5b	1.9b
PFA3	2208.3b	796.9bc	12.4b	1.8bc

a Note that means followed by the same lowercase letter within each column in the same year were not significantly different (*p* > 0.05) based on the analysis by one-way ANOVAs followed by Duncan multiple-range tests (*n* = 6).

**Figure 3:**
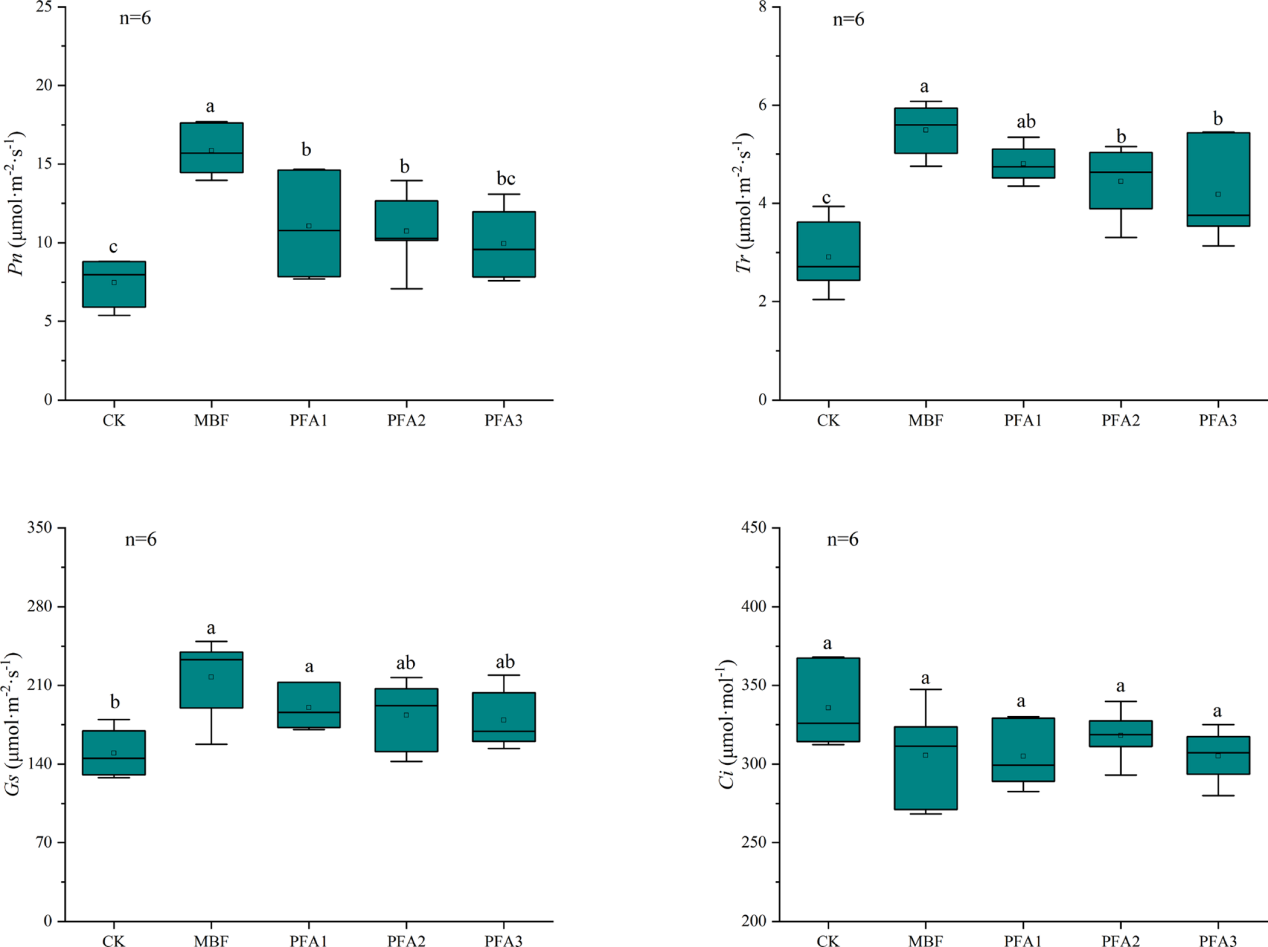
Photosynthetic characteristics of leaves under different treatments.

Photosynthesis is the basis of solar energy capture and nutrient accumulation that directly determines the plant productivity.^37^ The net photosynthetic rate (*Pn*) values in the MBF, PFA1, and PFA2 treatments were significantly increased by 112.0, 48.0, and 42.7%, respectively, compared with the CK treatment, whereas the *Pn* in the PFA3 treatment showed no significant difference with that in the CK treatment (Figure 3). Transpiration (*Tr*) was the main driving force of water/nutrient absorption and transportation.^38^*Tr* values in the MBF, PFA1, PFA2, and PFA3 treatments were 89.9, 65.5, 51.7, and 44.8%, respectively, significantly higher than those in the CK treatment, and no significant difference in *Tr* was observed between the PFA1 and MBF treatments. The increase in stomatal conductance (*Gs*) was helpful in promoting the gas exchange in blades. The *Gs* values in MBF and PFA1 treatments were 45.0 and 27.0%, respectively, higher than that in the CK treatment, but no significant difference in *Gs* was observed between different PFA dosages. In addition, there was no convincing evidence that PFA had a significant effect on the intercellular CO_2_ concentration (*Ci*) in leaves. Moreover, PFA may induce the abundance of certain proteins, thereby delaying the senescence of leaves caused by stress.^39^

**Figure 4:**
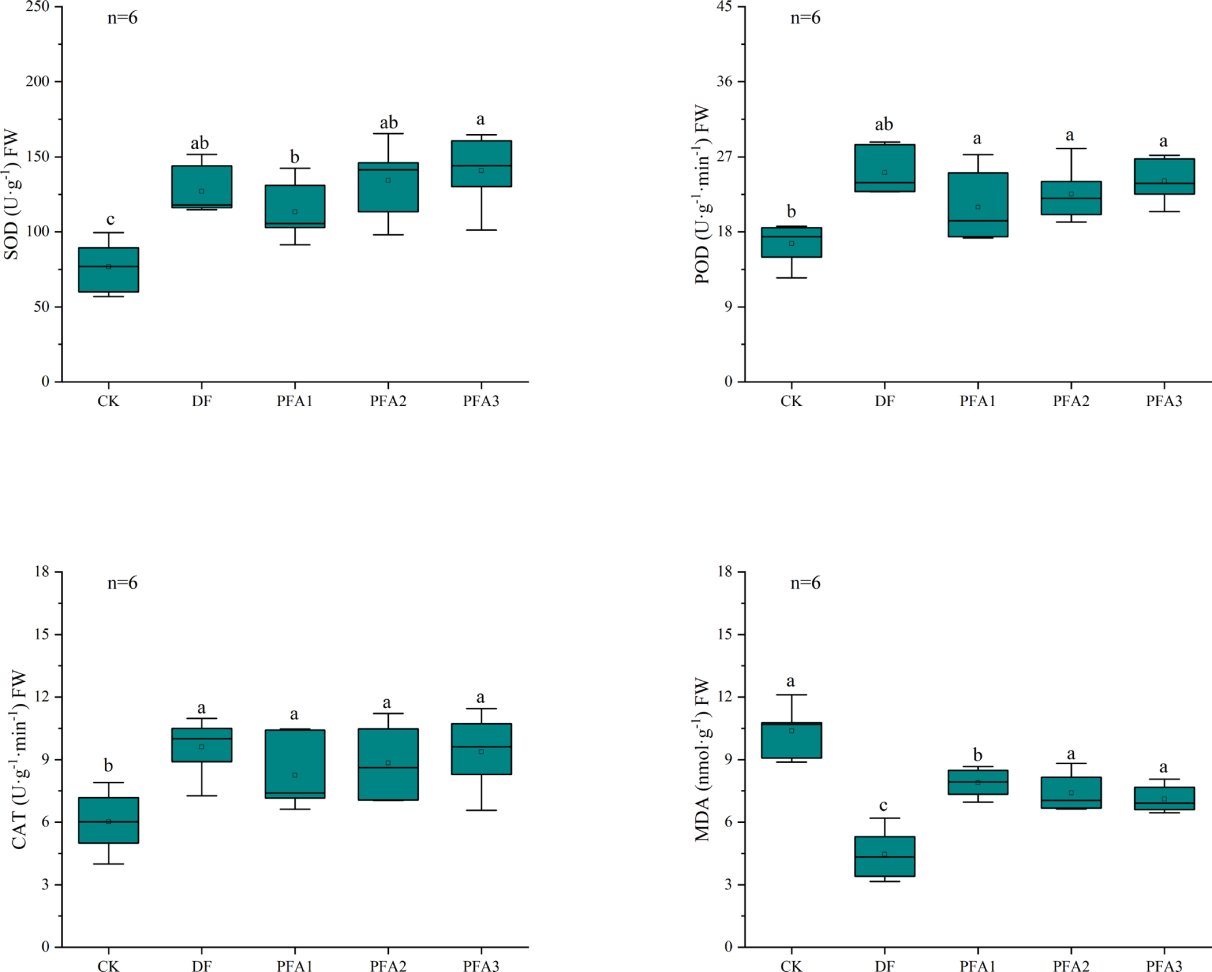
SOD, POD, and CAT enzyme activity and MDA contents of roots under different treatments.

Damaged plants will continually produce reactive oxygen species (ROS) including H_2_O_2_ and O_2_^–^ under adverse conditions, thereby seriously damaging the biological macromolecules of nucleic acids and proteins.^40,41^ Antioxidant enzymes, represented by superoxide dismutase (SOD), peroxidase (POD), and catalase (CAT), are conducive to removing ROS and improving plant resistance to stress.^22^ In the present study, all methyl bromide fumigation or PFA application treatments resulted in significantly higher SOD, POD, and CAT enzyme activity than that in the untreated CK (Figure 4), thereby reducing cell oxidative damage and enabling the normal growth of roots. With the increase in PFA dosage, the activity of the SOD, POD, and CAT enzymes showed an upward trend, and SOD in PFA3 was 24.5% significantly higher compared with that in the PFA1 treatment. The ROS produced under adverse conditions can trigger membrane lipid peroxidation. Malondialdehyde (MDA) is the final product of lipid peroxidation with cytotoxicity that can give rise to secondary damage to plants.^42^ The MDA content in CK was significantly higher than that in the treatments with fumigation or PFA, which partly confirmed that the level of damage of the CK treatment was more serious. In addition, the MDA content in the PFA application treatments was significantly higher than that in the MBF treatment, but no significant difference was observed between the treatments with different PFA dosages.

Under continuous cropping conditions, the roots directly contacting with soil will be damaged first; subsequently, the damaged roots will affect the absorption of water and nutrients and further interfere with the photosynthesis, respiration, and other life processes of plants. PFA can stimulate nutrient uptake and boost root growth under stress by inducing the activity of H^+^-ATPase and its subsequent effect on energizing secondary ion transporters.^22,43^ Moreover, Yao *et al*.^23^ also pointed out that carboxyl and phenolic hydroxyl groups in PFA were active functional groups with biostimulatory activity that is conducive to enhance the stress resistance of plants. In this experiment, the application of PFA markedly improved the root development and antioxidant enzyme activity of *M. hupehensis* Rehd. seedlings, and its positive effects on root growth were also transformed and manifested in leaves, thus partly affecting the photosynthetic capacity and gas exchange of leaves. A previous study has claimed that PFA has no significant effect on the *Pn* of plant leaves.^44^ However, in this study, the *Pn* and *Tr* were significantly increased due to PFA application, which may be related to the alleviation of plant stress.

### 2.4 Effects of Different Dosages of PFA on the Quantity of Soil Microorganisms and *Fusarium* Fungi

Although the consistency and heterogeneity of ARD were questionable, an observed reduction of the typical symptoms of ARD in studies where pasteurization,^45^ gamma-radiation,^46^ heating,^6^ or fumigants^47^ were applied in diseased soils provided strong evidence for the role of biotic factors on the emergence of this disease. Methyl bromide fumigation indiscriminately killed most microorganisms in soil; therefore, the numbers of bacteria and fungi in MBF were significantly lower than those in other treatments without fumigation (Table 4). The superior growth status of seedlings in the MBF treatment further confirmed the previous conclusion as noted above. However, no significant associations were detected in the numbers of soil bacteria and fungi with different PFA applications. The ratio of bacteria to fungi is commonly used as an important indicator reflecting the level of soil health. In the present experiment, the ratio of bacteria to fungi in MBF treatment was significantly higher than that in other treatments after soil microorganisms were recovered from fumigation, whereas no significant difference in the ratio of bacteria to fungi was observed under different PFA doses.

**Table 4:** Bacteria Amounts, Fungi Amounts, and Bacteria/Fungi Ratio under Different Treatments^a^

treatment	bacteria amounts (10^5^ CFU g^–1^ soil)	fungi amounts (10^3^ CFU g^–1^ soil)	bacteria/fungi ratio
CK	8.7a	16.4a	55.4b
MBF	2.1b	2.8b	77.3a
PFA1	8.5a	15.6a	56.8b
PFA2	8.4a	15.8a	53.9b
PFA3	8.6a	14.9a	59.1b

a Note that means followed by the same lowercase letter within each column in the same year were not significantly different (*p* > 0.05) based on the analysis by one-way ANOVAs followed by Duncan multiple-range tests (*n* = 6).

The causal agents of ARD varied from site to site and region to region where major pathogenic fungi for ARD including *Cylindrocarpon*,^48^*Rhizoctonia*,^49^*Phytophthora*,^50^*Pythium*,^51^ and *Fusarium*(52) have been discovered in the main apple-producing areas worldwide. In China, field investigations in the main apple-producing areas have found *Fusarium oxysporum*, *Fusarium solani*, *Fusarium proliferatum*, and *Fusarium moniliforme* to be the main pathogenic fungi causing ARD.^53,54^ In a study by Wang *et al.*,^20^ it was found that certain biostimulants, such as chitin, exhibited remarkable effects on soil microorganisms, thus significantly improving the growth of replanted seedlings. Moreover, another research claimed that the chemical composition of humic substances may be suitable to behave as a carrier to introduce beneficial microorganisms into cropping systems.^43^ However, in this study, the addition of different PFA dosages did not significantly change the copy number of the major pathogenic *Fusarium* fungi (Table 5). Since the microbial community structure is the decisive factor for its ecological functional characteristics and strength, thus, it is necessary to investigate the effects of PFA on the soil microbial community structure in the future.

**Table 5:** qRT-PCR Analysis of *F. oxysporum*, *F. solani*, *F. proliferatum*, and *F. moniliforme*^a^

treatment	*F. oxysporum* (10^6^)	*F. solani* (10^6^)	*F. proliferatum* (10^6^)	*F. moniliforme* (10^6^)
CK	6.7a	8.0a	7.8a	5.9a
MBF	2.3b	2.9b	2.7b	1.9b
PFA1	6.2a	8.1a	7.5a	5.9a
PFA2	6.3a	7.5a	7.1a	5.2a
PFA3	6.0a	8.1a	7.4a	5.5a

a Note that means followed by the same lowercase letter within each column in the same year were not significantly different (*p* > 0.05) based on the analysis by one-way ANOVAs followed by Duncan multiple-range tests (*n* = 6).

### 2.5 Correlation Analysis of the Aboveground Biomass and Root Weight of the Replanted Seedlings with Soil, Plants, and Soil Microorganisms

**Figure 5:**
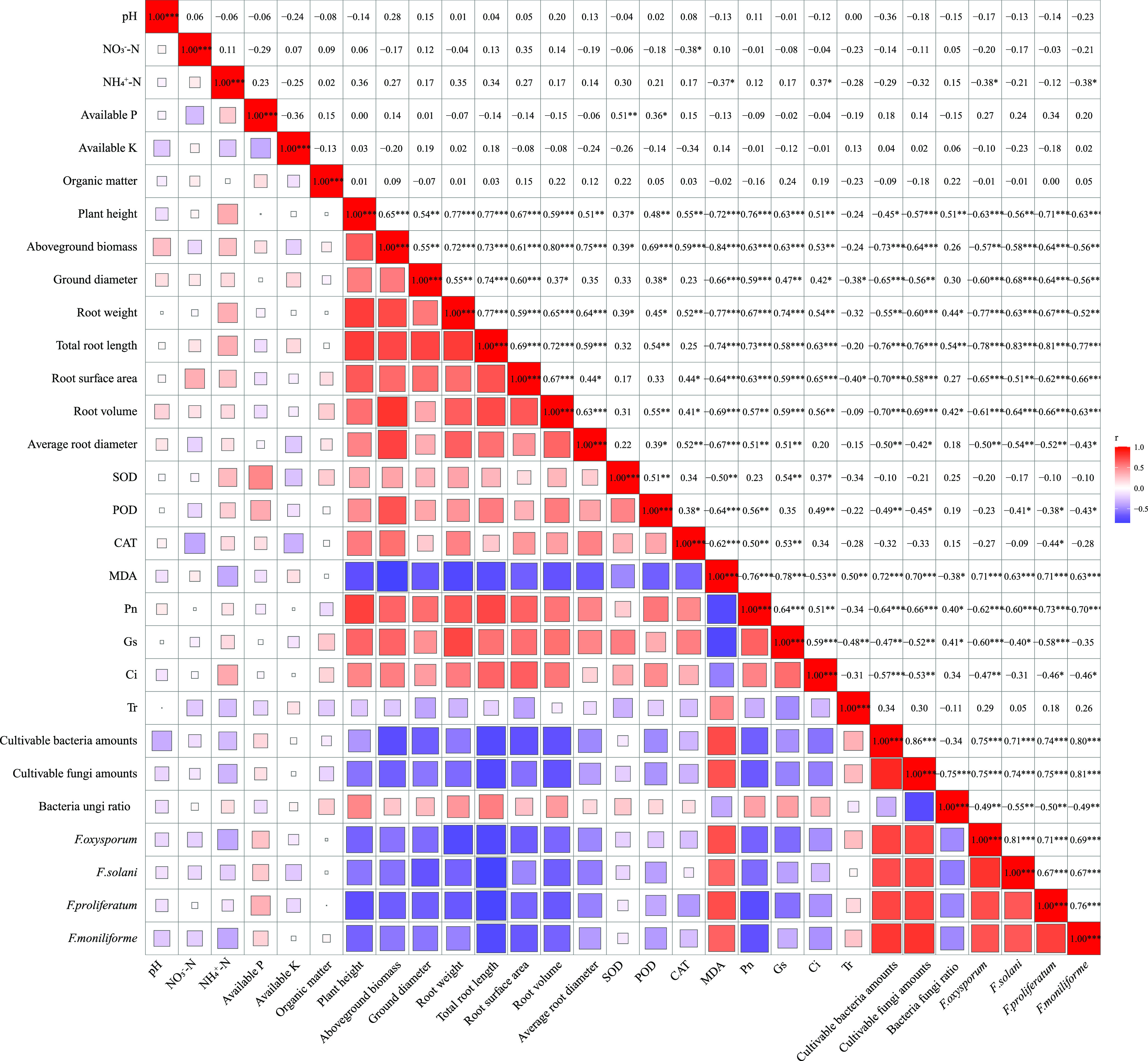
Correlation analysis of plant/soil properties and soil microorganisms among all treatments based on Pearson’s correlation analysis.

**Figure 6:**
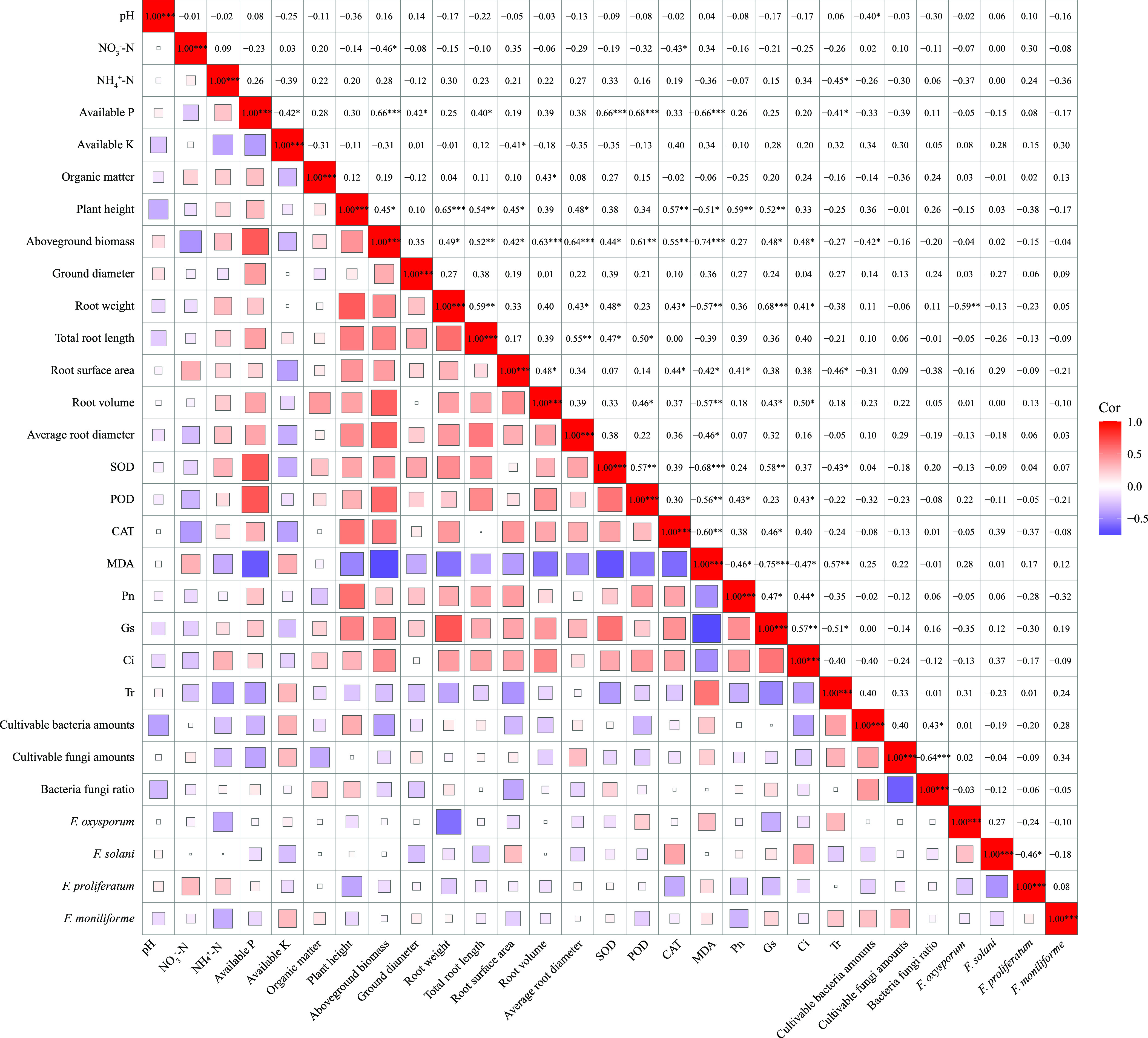
Correlation analysis of plant/soil properties and soil microorganisms in the treatments with different PFA dosages (CK, PFA1, PFA2, and PFA3) based on Pearson’s correlation analysis.

Simply put, this study confirmed that different doses of PFA were beneficial for alleviating ARD, even though it failed to have the same effect as methyl bromide fumigation. Subsequently, since the aboveground biomass and root weight were the most intuitive indicators for judging the severity of ARD, correlation analyses of the soil properties, plant growth status, and soil microorganisms with the aboveground biomass and root weight of the replanted seedlings were performed using Pearson’s correlation analysis. The results on all treatments showed that there was a negative correlation between the aboveground biomass/root weight and pathogenic *Fusarium*/MDA content (Figure 5). Moreover, the results of the treatments with different PFA doses indicated that the effects of PFA on the soil properties and microorganisms had no obvious correlation with the aboveground biomass and root weight of the replanted seedlings, whereas aboveground biomass and root weight were mainly determined by the growth status of the plant (Figure 6). This finding partly suggested that the ability of PFA to boost plant stress resistance mainly stemmed from its stimulation rather than the provision of nutrients, which precisely supports the idea that the mechanism of biostimulants in increasing crop yields is mainly through the regulation of the physiological and biochemical responses of plants rather than by the provision of nutrients for plants.^17,19^

**Figure 7:**
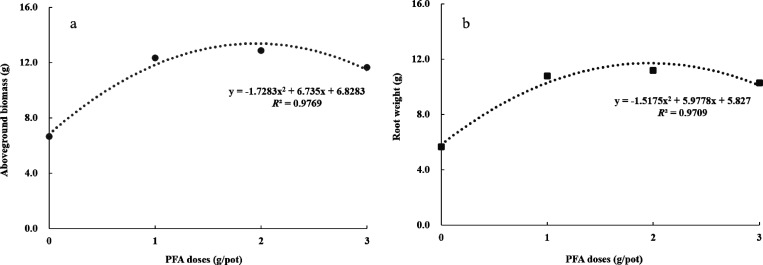
Correlation analysis of aboveground biomass and PFA doses (a) and root weights and PFA doses (b).

Subsequently, the correlation between the aboveground biomass and root weight of replanted seedlings and the PFA doses was analyzed. The results revealed that the aboveground biomass and root weight of the replanted seedlings increased at first and then decreased with increasing dosage of PFA. More specifically, the aboveground biomass (*y*_1_) and PFA doses (*x*) displayed a parabolic trend of *y*_1_ = −1.7283*x*^2^ + 6.735*x* + 6.8283 (*R*^2^ = 0.9769), and the maximum aboveground biomass appeared when PFA was applied at 1.95 g/pot (Figure 7a). Similarly, the root weight (*y*_2_) and PFA doses exhibited a parabolic trend of *y*_2_ = −1.5175*x*^2^ + 5.9778*x* + 5.827 (*R*^2^ = 0.9709), and the maximum root weight appeared at 1.97 g/pot (Figure 7b). Overall, the best dosage of PFA was roughly 2 g/pot, whereas it showed a decreasing trend when PFA was applied at 3 g/pot. Similar conclusions on FAs were also reached by other researchers that strong induction of a defense response is often accompanied by growth inhibition.^32,55^

In the future, an integrated approach in ARD control will be more extensive in view of the complex pathogenesis cause of ARD, the diversity of pathogenic microorganisms, and the differences in soil properties. A growing number of researchers have concentrated on the study of synergistic effects of rootstocks, soil disinfestation/fumigation, and biorenovation (using *Brassica*/mustard seed meals).^13,14,56^ The results of our study showed that PFA had no bactericidal and bacteriostatic effects, but it had strong biostimulatory effects on improving plant stress resistance. Therefore, PFA can be used in combination with fumigant/microbial agents, thus regulating soil microorganisms, stimulating plant growth, and enhancing plant stress resistance, to achieve the healthy growth of the replanted young trees. Previous research also convinced that the use of biostimulants in combination with pesticides improved the action of pesticides and then reduce the used rates of pesticides.^57^ In other words, the application of biostimulants can be considered as an important candidate/supplement in ARD control; especially, much attention is mainly focused on soil microbiota health.

## 3 Conclusions

Application of PFA-activated soil-available P promoted the growth of replanted *M. hupehensis* Rehd. seedlings through its stimulation in promoting root elongation, boosting the activity of root antioxidant enzymes, and enhancing leaf photosynthesis. However, this positive process did not seem to be associated with the numbers of soil bacteria and fungi as well as the main pathogenic *Fusarium*.

## 4 Materials and Methods

### 4.1 Experimental Sites and Materials

**Figure 8:**
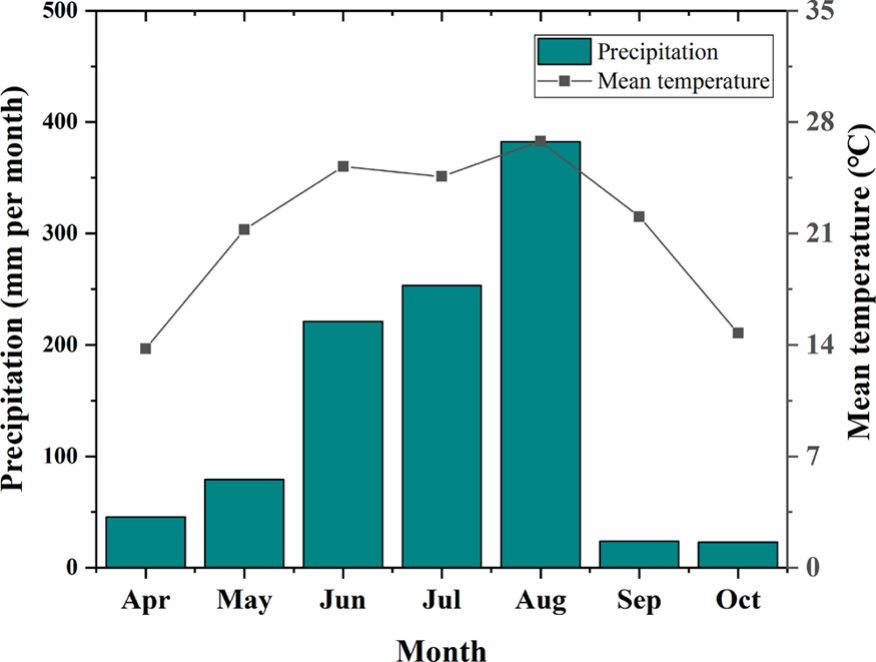
Monthly mean temperatures and total precipitation at the experimental site from April to October in 2021.

This pot experiment was conducted in the experimental station of the National Apple Engineering Technology Research Center on the south campus (36°09′16″ N, 117°09′01″ E) of Shandong Agricultural University from March 2020 to November 2020. Monthly mean temperatures and total precipitation during April to October are described in Figure 8. The soil used in the experiment was collected from an old apple orchard at Manzhuang, Tai’an city, Shandong Province, China, which has 25 years of cropping history of continuous apple growth. Basic properties of the soil before seedlings were planted were as follows: pH 6.8; organic matter, 7.2 g/kg; NO_3_^–^-N, 16.3 mg/kg; NH_4_^+^-N, 8.5 mg/kg; available P, 11.2 mg/kg; available K, 91.2 mg/kg. PFA was extracted from paper mill effluent produced by the straw-based ammonium sulfite pulping process,^23^ which was freely provided by Tranlin Co., Ltd. (Shandong, China). In addition, the pot used in the experiment has an upper diameter of 25 cm, a lower diameter of 17 cm, and a height of 18 cm.

### 4.2 Experimental Design

There were five treatments with six replicates in the present experiment. Treatments included the following: (1) continuous cropping soil (CK); (2) continuous cropping soil fumigated with methyl bromide (MBF); (3) continuous cropping soil applied with PFA at 1 g/pot (PFA1); (4) continuous cropping soil applied with PFA at 2 g/pot (PFA2); (5) continuous cropping soil applied with PFA at 3 g/pot (PFA3). Soil (6.5 kg) and PFA were homogeneously mixed prior to the replanting of seedlings.

The commonly used rootstock, *M. hupehensis* Rehd., was selected as the indicator plant. Stratified seeds were germinated in a growth chamber at 4 °C for 30 days. When the seedlings were 2 months old with six true leaves, two prepared seedlings of uniform size were replanted in the pot on 9 June 2020. All replications within each treatment were conducted following the usual practices, receiving identical irrigation, pruning, and control of insects and weeds.

### 4.3 Characterization of PFA

Gel permeation chromatography (GPC) was performed on water solutions of the sample using a chromatograph (LC 20, Shimadzu, Japan). Fourier transform infrared (FTIR) spectra were recorded with a spectrometer (Nicolet IS 10, Thermo Fisher, America) over the 4000–400 cm^–1^ range, with a resolution of 0.4 cm^–1^. Solid-state ^13^C NMR spectroscopy and liquid-state ^1^H NMR spectroscopy of PFA were conducted with a spectrometer (Bruker AVANCE III 600 M, Karlsruhe, Germany).

### 4.4 Sample Collection and Analysis

Soil, plants, and soil microorganisms were collected on 14 November 2020.

Plant height and ground diameter were measured by a ruler. The photosynthesis parameters in leaves including the net photosynthetic rate (*Pn*), stomatal conductance (*Gs*), intercellular CO_2_ concentration (*Ci*), and transpiration (*Tr*) were measured from 9 to 11 am by a portable photosynthetic meter (Li-6400XT, LI-COR, America). Subsequently, the seedlings were pulled out of the soil and taken back to the laboratory. Six seedlings (one seedling per pot) were used to measure the root total length, root surface area, root volume, and average diameter by a root analysis imaging system (LA-S, Wseen, China). After the root determination analysis, the roots were air-dried at 60 °C to a constant weight and used to measure the aboveground biomass and root weight. Moreover, other six seedlings were used to measure the activity of superoxide dismutase (SOD), peroxidase (POD), catalase (CAT), and malondialdehyde (MDA) contents following Chen *et al.*’s method.^58^

The soil sample in each pot was divided into two parts: one part was used to determine NO_3_^–^-N and NH_4_^+^-N in a fresh sample immediately, and the other part was air-dried and ground to pass through a sieve for the further analysis. pH (ratio of soil/water, 1:2.5) was measured by a pH meter (PB-10, Sartorius, Germany). NO_3_^–^-N and NH_4_^+^-N were extracted by 0.01 M CaCl_2_^17^ and analyzed by the AA3 Auto-analyzer (model AA3-A001-02E, Bran-Luebbe, Germany). Available P was extracted by 0.5 M NaHCO_3_ at pH 8.5^17^ and analyzed by the Discrete Auto-analyzer (Smart Chem 200, Alliance, France). Available potassium (K) was extracted by 1 M CH_3_COONH_4_ at pH 7^17^ and analyzed by the flame photometer (Model 410, Sherwood, England). Soil organic matter was measured according to the K_2_Cr_2_O_7_-H_2_SO_4_ oxidation method.^59^

The soil within 2 mm of the root surface was recognized as the rhizosphere samples. Roots of the seedlings were pulled out of pot; subsequently, rhizosphere samples were carefully collected by a sterile brush.^60^ Soil bacteria and fungi were determined according to the dilution plate counting method. More specifically, soil bacteria were cultured using a beef extract peptone medium at 37 °C, while soil fungi were cultured using PDA selective medium at 28 °C.^58^ Copy numbers of *F. oxysporum*, *F. solani*, *F. proliferatum*, and *F. moniliforme* were measured using real-time quantitative PCR (qPCR) according to the study by Wang *et al.*(53)

### 4.5 Data Analysis

The response parameters were subjected to the analysis of variance (ANOVA) and mean separation test using the Statistical Analysis System package version 9.2 (2010, SAS Institute Cary, NC). Associations among plants, soil, and microbes were assessed using Pearson’s correlation analysis. Means and standard error values were assessed to assemble graphs using SigmaPlot software version 10 (MMIV Systat Software, Inc., San Jose, CA).
